# Mevalonate-derived quinonemethide triterpenoid from *in vitro* roots of *Peritassa laevigata* and their localization in root tissue by MALDI imaging

**DOI:** 10.1038/srep22627

**Published:** 2016-03-04

**Authors:** Edieidia S. Pina, Denise B. Silva, Simone P. Teixeira, Juliana S. Coppede, Maysa Furlan, Suzelei C. França, Norberto P. Lopes, Ana Maria S. Pereira, Adriana A. Lopes

**Affiliations:** 1 Unidade de Biotecnologia, Universidade de Ribeirão Preto, Av. Costábile Romano, 2201, 14096-900, Ribeirão Preto, SP, Brazil; 2 Núcleo de Pesquisa em Produtos Naturais e Sintéticos (NPPNS), Faculdade de Ciências Farmacêuticas de Ribeirão Preto, Universidade de São Paulo, Ribeirão Preto, SP, 14040-903, Brazil; 3 Universidade Federal de Mato Grosso do Sul (UFMS), Laboratório de Produtos Naturais e Espectrometria de Massas (LAPNEM), Campo Grande, MS, 79070-900, Brazil; 4 Faculdade de Ciências Farmacêuticas de Ribeirão Preto, Laboratório de Botânica, Universidade de São Paulo, Ribeirão Preto, SP, 14040-903, Brazil; 5 Instituto de Química, Universidade Estadual Paulista, Araraquara, SP, 14801-970, Brazil

## Abstract

Biosynthetic investigation of quinonemethide triterpenoid 22β-hydroxy-maytenin (**2**) from *in vitro* root cultures of *Peritassa laevigata* (Celastraceae) was conducted using ^13^C-precursor. The mevalonate pathway in *P. laevigata* is responsible for the synthesis of the quinonemethide triterpenoid scaffold. Moreover, anatomical analysis of *P. laevigata* roots cultured *in vitro* and *in situ* showed the presence of 22β-hydroxy-maytenin (**2**) and maytenin (**1**) in the tissues from transverse or longitudinal sections with an intense orange color. MALDI-MS imaging confirmed the distribution of (**2**) and (**1**) in the more distal portions of the root cap, the outer cell layers, and near the vascular cylinder of *P. laevigata in vitro* roots suggesting a role in plant defense against infection by microorganisms as well as in the root exudation processes.

The metabolic engineering of plants has been a relevant biotechnological tool for the production of secondary metabolites^1^. Root cultures can provide an alternative approach for producing important phytochemicals, as well as for understanding their biosynthetic pathways^2^. Camptothecin, vinblastine and ginsenosides are examples of important secondary metabolites stored in roots^3^,^4^. Hence, roots have been studied to induce and culture *in vitro* systems, such as adventitious root cultures, that are not infected with *Agrobacterium rhizogenes*^2^. Adventitious roots are natural and genetically well-established organs, in contrast to cultured plant cell suspensions, and they are useful for biosynthetic investigation as well as for biotechnological applications^5^,^6^. Cytotoxic triterpenoids that accumulate in adventitious roots are of great interest due to their extensive range of biological activity, especially in their potential effects against human tumor cell lines^7^,^8^. Recently, a subclass of terpenoids, namely quinonemethide triterpenoids, has been found to display notable antitumor activity against myeloma cell proliferation^9^, hepatocellular carcinoma cells^10^, prostate cancer cells^11^, glioblastoma cells^12^ and pancreatic cancer cells^13^. A terpenoid is synthesized in nature through one of two pathways involved in the biosynthesis of isoprene units (IPP), the mevalonate (MVA) and methylerythritol phosphate (MEP) pathways^14^. Both the MVA and MEP pathways are localized in individual cellular compartments: the MVA pathway is located in the cytosol and the MEP pathway in the plastids^15^. The MVA pathway has long been known for triterpene biosynthesis^16^ and despite several studies on the MEP pathway, its complex regulation mechanism has not yet been fully clarified^14^. Although quinonemethide triterpenoid biosynthesis has not yet been investigated, there is one report on how mevalonic acid is responsible for IPP building blocks and also that friedelin (friedelane triterpenoid) is a primary precursor of maytenin and pristimerin^17^.

Here, we show for the first time the complete biosynthetic origin of the IPP units in maytenin (**1**) and 22β-hydroxy-maytenin (**2**) accumulated in *in vitro* root cultures of *Peritassa laevigata* (Hoffmanns. ex Link) A.C.Sm. using the incorporation of 1-^13^C-D-glucose as a ^13^C-labeled precursor. Besides, we carried out anatomical analysis from *in vitro* root and *in situ* root cultures and MALDI imaging from *in vitro* roots to localize the compartmentalization of compounds **1** and **2** in root cells.

## Results

### Quantification of 1 and 2 from *in vitro* and *in situ* root cultures of *P. laevigata*

**Figure 1:**
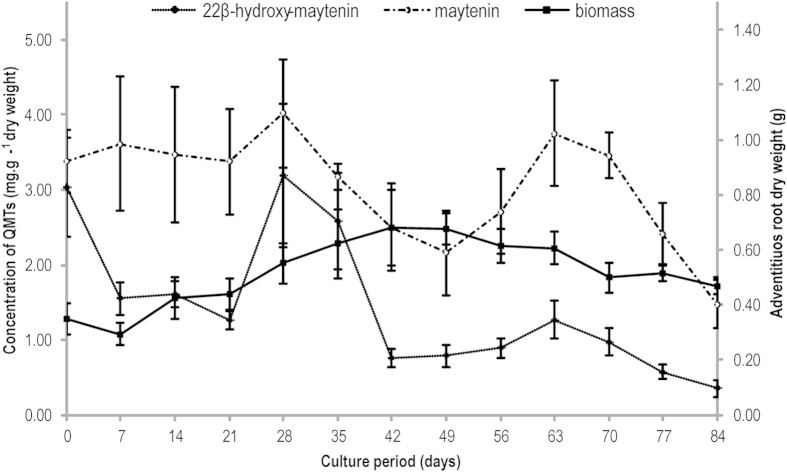
Quantification of maytenin (1) and 22β-hydroxy-maytenin (2) in *P. laevigata* adventitious roots cultured *in vitro* (dry weight). The roots were cultured for 84 days under dark conditions in WPM medium supplemented with 2% glucose (w/v), PVP (899.74 μM), and IBA (19.68 μM).

*In vitro* root cultures were established from the cotyledon of *P. laevigata*. Maytenin (**1**) and 22β-hydroxy-maytenin (**2**) were the major compounds found in *in vitro* roots from *P. laevigata*, and there is no previous study in the literature reporting its chemical composition. Higher accumulation of quinonemethide triterpenes [4.02 ± 0.72 mg.g^−1^ of **1** and 3.20 ± 0.95 mg.g^−1^ of **2**] occurred at 28 days for *in vitro* roots cultivated from *P. laevigata* (Fig. 1). *In situ* roots from *P. laevigata* accumulated 7.76 ± 0.02 mg.g^−1^ of **1** and 0.47 ± 0.08 mg.g^−1^ of **2** in a 3-year-old cultivation, while roots from ten-year-old plants cultured *in situ* produced 8.54 ± 0.95 mg.g^−1^ of **1** and 0.54 ± 0.04 mg.g^−1^ of **2** in a 10-year-old cultivation (Table 1).

### Biosynthetic origin of quinonemethide triterpenoids from *P. laevigata* roots cultured *in vitro*

**Figure 2:**
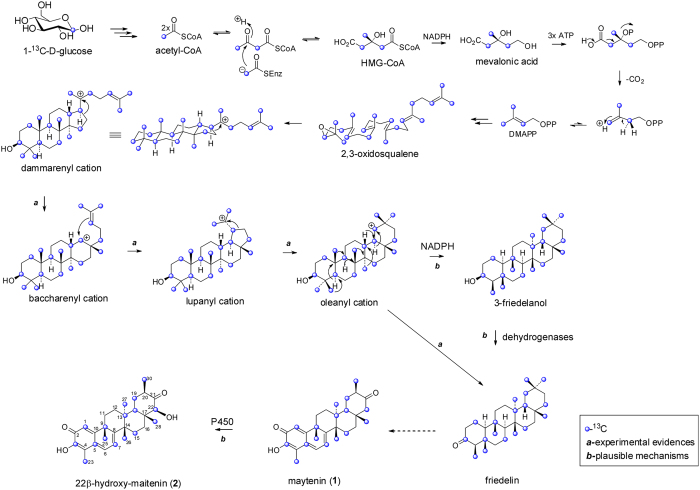
Biosynthetic studies using 1-^13^C-D-glucose as precursor showed that the biosynthesis of quinonemethide triterpenoids **1** and **2** proceeds via mevalonate pathway.

To observe the biosynthetic pathway of the target quinonemethide triterpenoids, *in vitro* roots were cultured in Murashige & Skoog medium^18^ supplemented with 1-D-^13^C-glucose precursor for 30 days. A chloroform extract from fresh root cultures of *P. laevigata* was then prepared and fractioned by column chromatography to yield **2**. The incorporation pattern was determined by quantitative ^13^C NMR by comparing the relative intensities of the labeled and non-labeled signals for **2**. After 1-^13^C-D-glucose metabolism, the ^13^C-enrichment pattern of **2** showed that the positions C-1, C-3, C-5, C-7, C-9, C-13, C-15, C-18, C-19, C-22, C-23, C-25, C-26, C-27, C-28 and C-30 (Fig. S2, Table S1 - Supplementary Information) were highly labeled with ^13^C (3.1% to 6.3% range). The MVA pathway generates an IPP unit enriched in C-2, C-4 and C-5 while an IPP unit from the MEP pathway is enriched in C-1 and C-5. Obtained data confirm that the IPP building units were biosynthesized exclusively by the MVA pathway since quinonamethide triterpenes are biosynthesized by 6 IPP units, and therefore 18 C-positions would be labeled, however only 16 C-positions labeled were found (Fig. 2 and Table S1- Supplementary Information). A hypothesis could be that two methyl groups undergo further descarboxylation reaction (Fig. S3-Supplementary Information). The initial precursor *chair–chair–chair–boat* conformation of 2,3-oxidosqualene undergoes a series of Wagner–Meerwein rearrangements, first hydride migration generating a new cation followed by 1,2-methyl rearrangement^19^. The dammarenyl cation (tertiary cation) then undergoes ring expansion, giving the baccharenyl cation. The baccharenyl cation is converted to a 5-membered ring followed by the formation of the tertiary lupanyl carbocation. Wagner–Meerwein 1,2-methyl rearrangement of the lupanyl cation occurs, leading to an oleanyl cation^19^. The oleanyl cation is converted to friedelin, a key precursor of quinonemethide triterpenes^20^. Regarding friedelin, the hypothetical pathway also involves sequential oxidations, most likely by cytochrome P450 enzymes, which may catalyze more than one oxidation reaction leading to intermediates such as celastrol and maytenin (**1**). Then, maytenin (**1**) is converted to 22β-hydroxy-maytenin (**2**) through one stereospecific hydroxylation at position C-22, and the presence of both is confirmed in root cultures (Fig. 2 and Fig. S3-Supplementary Information).

### Anatomical studies of *P. laevigata* roots

It has been reported that quinonemethide triterpenoids are produced and accumulate in roots of several Celastraceae species^17^, and understanding their unexplored biosynthesis is a fundamental step to manipulate their production. In addition, their tissue distribution, which has not yet been investigated, could contribute to identify their functions in the tissue and in root the exudation process^21^, and also to improve biotechnological manipulation. The *in vitro* root culture showed typical root anatomy (Fig. S4-S9 Supplementary Information). Numerous black starch grains were observed in cells of cortical parenchyma from *in vitro* roots (Fig. S4A-B Supplementary Information), similar to that observed for *ex vitro* roots (Fig. S4C-D Supplementary Information) when stained with Lugol, and the shapes of starch grains were visualized by polarized light, as observed for *in vitro* (Fig. S5A Supplementary Information) and *in situ* roots (Fig. S5B Supplementary Information). Transversal sections of *in vitro* and *in situ* roots without dye stabilization showed an accumulation of orange color compounds detected in endoderm and cortical parenchyma cells from *in vitro* (Fig. S6A,C Supplementary Information) and *in situ* roots (Fig. S6B,D Supplementary Information), suggesting the compartmentalization of compounds **1** and **2**, as already reported for *in vitro* roots of *Peritassa campestris* that substances that have been isolated have an intense orange color^22^,^23^,^24^,^25^. The longitudinal sections of *in vitro* culture roots (Fig. S9 Supplementary Information), with no stain applied, showed more intense orange color in the region of root primary structure (Fig. S9C Supplementary Information) compared to near to root cap (Fig. S9A Supplementary Information), which denotes a possible higher accumulation of orange color compounds in outer layer cells. Subsequently their chemical identification and tissue distribution were confirmed by MALDI imaging. In addition, the transversal (Fig. S7A,B Supplementary Information) and longitudinal (Fig. S7C, Fig. S8A–C Supplementary Information) sections of *in vitro* roots were stained with safranine/astra blue and the distributions of the primary and secondary cell walls were determined in the tissue, confirming its common root anatomy. Details of xylematic elements stained pink using safranin/astra blue dye could be observed by longitudinal sections (Fig. S7C Supplementary Information).

### Compartmentalization and tissue distribution of quinonemethide triterpenoids (1–2) of *in vitro* root cultures from *P. laevigata* by MALDI imaging

**Figure 3:**
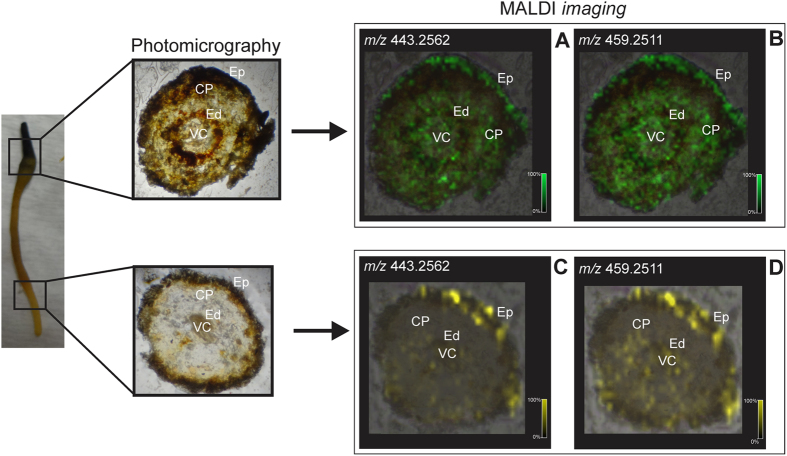
Transverse sections of root tissues from *Peritassa laevigata* obtained from different parts (root cap, differentiating region, and primary structure) and their MALDI-MS images reconstructed with ions m/z 443.2562 [M + Na]^+^ (**A,C**) and 459.2511 [M + Na]^+^ (**B,D**) corresponding to maytenin (**1**) and 22β-hydroxy-maytenin (**2**), respectively. CP: cortical parenchyma; Ed: endoderm; Ep: epiderm; VC: vascular cylinder.

The identity and distribution of compounds **1** and **2** was confirmed by MALDI imaging, an emerging technique for visualization of metabolite distributions in plant tissues^26^,^27^,^28^,^29^. Firstly, the standards were analyzed by MALDI-MS and MS/MS and it was possible to detect their sodiated ions and a reduction of source fragmentation after sodium addition. Subsequently, transverse sections of *in vitro* roots were analyzed by MALDI imaging. The images reconstructed with ions of **1** (*m/z* 443.2562 [M + Na]^+^) and **2** (*m/z* 459.2511 [M + Na]^+^), which showed higher ion intensities in the distal portions than near the root cap, demonstrated higher accumulation in older tissues (Fig. 3, Fig. S10 Supplementary Information). Moreover, higher ion intensity was observed in the endoderm and outer cell layers (Fig. 3A,B), but they were extensively detectable in tissues such as the cortical parenchyma.

## Discussion

Unlike most plant species, the maximum accumulation of quinonemethide triterpenes in *P. laevigata in vitro* roots preceded biomass enhancement, corroborating the results obtained with Celastraceae species cultured *in vitro*^30^,^31^,^32^. *P. laevigata* roots cultured *in vitro* produced 1.3 times more compound **2** compared to roots of 10-year-old plants cultured *in situ*, although yields of compound **1** was 3.3 fold higher in *P. laevigata* roots grown *in situ*. Our data suggest that sub-cultivation of roots could be highly advantageous, considering that roots cultured *in vitro* for four months produced the same amount of **1** and five times more the amount of **2** compared to the production in roots of 10-year-old plants cultured *in situ*. There is no statistical significance between the quinonemethide triterpenes amount of 3-year-old and 10-year-old *in situ* roots. Moreover, the production of **2** in *P. laevigata* roots cultured *in vitro* was superior compared to other Celastraceae species, including *Peritassa campestres* and *Maytenus ilicifolia*^30^,^31^,^32^. The cultivation of differentiated organs, such as root culture obtained from *P. laevigata*, can significantly improve the accumulation of secondary metabolites often considered cytotoxic, such as quinonemethide triterpenes, which are present in cells specialized for storage of compounds at specific stages of development. Compounds **1** and **2** could be related to protection against microorganisms, justifying their tissue distributions (higher ion intensities) in the outer cell layers and near the vascular cylinder, as they showed significant antimicrobial activity^8^,^33^, as well as facilitating the exudation processes toward growth medium, as observed in *Catharanthus roseus*^34^ and other species^21^. Roots are able to secrete defense compounds into the rhizosphere and this process is regulated by endogenous and exogenous stimuli. In fact, cap and border cells are involved in the development of roots and these cells act as a defensive barrier of roots protecting the plant against pathogen invasion^21^,^35^. More recently, it was reported that exudation of the isoflavonoid pisatin and the construction of the root border cell is stimulated in pea when root tips are challenged with a plant pathogen. Besides, exogenous pisatin leads to the upregulation of border cell production *in vitro*^35^. The production of antimicrobial naftoquinones and epidermal and outer layer cells increased, after the fungal elicitation in *Lithospermum erythrorhizon* roots^36^. Similar tissue distribution was observed in our study, and these findings together with the effective antimicrobial activity observed for maytenin and 22-β-hydroxy-maytenin^33^,^37^, suggest there are functions related to exudation and the antimicrobial protection. Altogether, our results regarding the biosynthesis of quinonemethide triterpenoids in *P. laevigata* have shown that they are constructed by the MVA pathway, and the route is compartmentalized in the cytosol. This study provides the first experimental evidence of quinonemethide triterpenoid biosynthesis using a ^13^C-precursor and shows the production and tissue distribution of quinonemethide triterpenoids in *P. laevigata* roots cultured *in vitro* using the MALDI imaging mass spectrometry. Obtained data present the possibility of developing large-scale production of quinonemethide triterpenoids, achieving greater levels than that produced by plants *in situ*, to supply the pharmaceutical industry with anticancer compounds and also to provide support for the manipulation of the biosynthesis of those compounds by biotechnological processes.

## Methods

### Chemical

The 1-^13^C-D-glucose was purchased from Sigma-Aldrich^®^. The matrices 2,5-dihydroxybenzoic acid (DHB) and α-cyano-4-hydroxycinnamic acid (CHCA) were purchased from Bruker Daltonics.

### Plant materials

The specimen was identified by Dr. Julio Antonio Lombardi (Instituto de Biociências, UNESP, Rio Claro, SP). A voucher specimen, under access number 2389, was deposited at the Herbarium of Medicinal Plants of the University of Ribeirão Preto (HPM-UNAERP, Ribeirão Preto, SP, Brazil). Seeds from *P. laevigata* were collected in the region of Água Limpa, MS, Brazil (November, 2010) and surface sterilized using a 1% captan solution (Orthocide^®^ 500) and thiophanate-methyl (Cercobin^®^ 700WP) for 24 h. After this period, seeds were transferred to a solution containing ampicillin, cefotaxime, gentamicin and streptomycin (10 mg.L^−1^ of each antibiotic) for another 24 h. Then, the seeds were immersed in calcium hypochlorite (0.5%) for 30 min, washed three times with distilled water and inoculated on semi-solid basal Murashige & Skoog medium supplemented with 30 g.L^−1^ sucrose and 2.5 g.L^−1^ Phytagel^®^. The explants were kept in a culture room at 25 ± 2 °C, 55–60% relative humidity under a 16/8 h photoperiod (light/dark). After 90 days of culture, cotyledons were excised and transferred to culture medium containing basal WPM medium supplemented with IBA (19.68 μM), PVP (899.74 μM), Phytagel^®^ (2.5 g.L^−1^) and glucose (20 g.L^−1^) and pH adjusted to 6.0, for root induction. Cotyledon segments presenting adventitious root formation were transferred to Erlenmeyer flasks containing the same culture medium described above (without Phytagel^®^) and placed on an orbital shaker (90 rpm) in the dark. *In vitro* root cultures were subcultured in a 60-day interval. Quinonemethide triterpenes **1** and **2** were quantified by biomass growth curves in the same culture medium used for root induction. For each sample, an initial inoculum of *P. laevigata* roots (2.00 ± 0.20 g) was placed into an Erlenmeyer flask (250 mL) containing 100 mL of culture medium and kept in growth chamber at 25 ± 2 °C, in the dark, on an orbital shaker under 90 rpm agitation. Sample root collection was performed for 84 days starting at day 0 (cultivation start). Roots cultured *in vitro* were removed from the culture medium (*n* = 9), weighed and dried in a circulating air oven for 48 h at 43 °C at seven-day intervals. Roots from 3-year-old (*n* = 3) and 10-year-old (*n* = 3) plants grown *in situ* collected from the field (UNAERP, Ribeirao Preto, SP - Brazil), were dried in circulating air oven for 48 h at 43 °C for seven days.

### General procedure for biosynthesis experiments from *P. laevigata* roots cultured *in vitro*

*In vitro* root cultures (twenty Erlenmeyers containing 3.0 g each) were maintained for a 60-day interval in basal WPM medium supplemented with IBA (19.68 μM), PVP (899.74 μM) and 1-D-^13^C-glucose (20 g.L^−1^) and pH adjusted to 6.0. *In vitro* roots (control) were cultivated with the same culture medium described above using D-glucose. The medium was able to induce younger roots after 2 weeks of culture under a cycle of 16 h light/8 h dark with continuous growth until 35 days.

### General procedure for the isolation of quinonemethide triterpenoids (1 and 2)

After 35 days, *in vitro* root cultures were extracted with chloroform for 14 h. The resulting extract (243.8 mg) was fractionated by column chromatography over silica gel (70–230 mesh; Merck, column size 9.0 × 2.0 cm) with *n*-hexane-EtOAc (7:3) as the mobile phase and with increasing amounts of EtOAc (up to 30%) to yield seven fractions. Fraction 2 (35 mg) was further purified via column chromatography over silica gel (70–230 mesh; Merck, column size 5.0 × 1.2 cm) using *n*-hexane-EtOAc (8:2) to yield **1** (5.0 mg). Fraction 5 (20 mg) was further purified via column chromatography over silica gel (70–230 mesh; Merck, column size 5.0 × 1.2 cm) using *n*-hexane-EtOAc (6:4) to yield **2** (8.0 mg).

### HPLC analysis

Dried plant material from roots cultured *in vitro* and *in situ* (1 g), was extracted with chloroform for 12 h to obtain the crude extract, and the sample preparation for quantitative HPLC analysis of **1** and **2** was performed as previously described by our group and colaborators^17^,^31^. HPLC analyses were carried out using the Shimadzu instrument (LC-10-AVP) system. The quantification of compounds **1** and **2** was performed on a Phenomenex - Luna (C-18) column 250 × 4.6 mm, 5 μ particle size, using a isocratic mobile phase, methanol/water/formic acid (80 : 20 : 0.1, v/v/v) for 20 min., 1.0 mL.min^−1^ flow rate; detection at 420 nm). Compounds **1** and **2** were detected at 9.22 min and 10.87 min, respectively, and the amount of compounds were calculated from a calibration curve (triplicate, prepared in acetonitrile at concentrations of 31.0, 63.0, 125.0, 250.0 and 500.0 μg.mL^−1^ for **1** and 7.81, 15.63, 31.25, 62.50 and 125.0 μg.mL^−1^ for **2**; Fig. S1 - Supplementary Information). The statistical analysis Scott-Knott test (P\0.05) was applied.

### NMR analysis

NMR spectra were recorded on a Bruker 400 MHz spectrometer using CDCl_3_ as the solvent and internal standard. The relative ^13^C enrichments were obtained by comparing the relative intensity of the labeled signal and the natural abundance of quinonemethide triterpenoids. The combination of 1D and 2D NMR experiments allowed complete elucidation consistent with the literature values^38^. All NMR spectra are described in the supplementary material.

### Sample preparation

The roots were transversely sectioned in a Leica RM2245 microtome at a thickness of 30 μm, and photomicrographs were obtained with a Leica DM 500 photomicroscope.

### MALDI imaging analyses

The MALDI imaging analyses were performed using a MALDI-TOF/TOF UltrafleXtreme (Bruker Daltonics, Bremen, Germany), equipped with an 1KHz smartbeam II laser and operating in reflectron positive ion mode. The transverse sections of roots were adhered with double-sided tape (3 M Co., USA) to indium tin oxide-coated conductive slides (Bruker Daltonics) for MALDI analysis. The matrix (DHB:CHCA 7:3 (w/w) was prepared at a concentration of 10 mg/mL with the addition of 0.15 mg/mL NaCl using acetonitrile and deionized water (9:1, v/v). The matrix was applied to the tissue by an ImagePrep station, and N_2_ flux was used in the entire spraying process. The instrumental conditions employed were as follows: ion source 1 of 25.00 kV, ion source 2 of 22.55 kV, pulsed ion extraction 110 ns, laser frequency 1000 Hz, minimum laser setting and 800 shots. The external calibration was performed using a flavonoid mixture (galangin, rutin, quercetin and isoquercetin). The images were collected at 25 μm spatial resolution in both the x and y directions. The spectra were calibrated internally using matrix ions and a corrected baseline. The tissue analysis of different parts of the roots (differentiating region and root primary structure) were analyzed together using different region of interest to compare the ion intensities and to apply the same processing parameters. The images were normalized, and a logarithm ion intensity scale was applied.

### Histochemical analyses

The root anatomy was analyzed in transverse and longitudinal sections stained with safranine/astra blue (safrablau) and lugol to detect starch and total lipids and terpenes into the root tissues, respectively. Lugol solution yielded a blue-black color in the presence of starch, while safrablau solution yielded a blue color in the presence of primary cell walls and a pink color for secondary cell walls (xylematic elements). Starch was also checked using polarized light. Unstained sections were also analyzed and photographed. All the images are presented in the supplementary material.

## Additional Information

**How to cite this article**: Pina, E. S. *et al*. Mevalonate-derived quinonemethide triterpenoid from *in vitro* roots of *Peritassa laevigata* and their localization in root tissue by MALDI imaging. *Sci. Rep*. **6**, 22627; doi: 10.1038/srep22627 (2016).

## Supplementary Material

Supplementary Information

## Figures and Tables

**Table 1:** Quantification of maytenin (1) and 22β-hydroxy-maytenin (2) in roots from 3-year-old and 10-year-old *P. laevigata* cultured *in situ*.

*In situ* roots	22β-hydroxy-maytenin (mg.g^−1^)	maytenin(mg.g^−1^)
3-year-old	0.47 a	7.76 a
10-year-old	0.54 a	8.54 a

Scott-Knott test (p < 0.05).
