# Supplementary material for: Mevalonate-derived quinonemethide triterpenoid from in vitro roots of Peritassa laevigata and their localization in root tissue by MALDI imaging
Source: Sci Rep. 2016 Mar 4;6:22627. doi: 10.1038/srep22627 (PMC4778575; doi:10.1038/srep22627)
Supplement: Supplementary Information [file srep22627-s1.docx]

**Supporting Information**

**Mevalonate-derived quinonemethide triterpenoid from *in vitro* roots of *Peritassa laevigata* and their localization in root tissue by MALDI imaging**

**Edieidia S. Pina^1^, Denise B. Silva^2,3^, Simone P. Teixeira^4^, Juliana S. Coppede^1^, Maysa Furlan^5^, Suzelei C. França^1^, Norberto P. Lopes^2,*^, Ana Maria S. Pereira^1^ and Adriana A. Lopes^1,*^**

*^1^ Unidade de Biotecnologia, Universidade de Ribeirão Preto, Av. Costábile Romano, 2201, 14096-900, Ribeirão Preto, SP, Brazil.*

^2^ *Núcleo de Pesquisa em Produtos Naturais e Sintéticos, Faculdade de Ciências Farmacêuticas de Ribeirão Preto, Universidade de São Paulo, Ribeirão Preto, SP, 14040-903, Brazil.*

*^3^Universidade Federal de Mato Grosso do Sul (UFMS), Laboratório de Produtos Naturais e Espectrometria de Massas (LAPNEM), Campo Grande, MS, 79070-900, Brazil.*

*^4^Faculdade de Ciências Farmacêuticas de Ribeirão Preto, Laboratório de Botânica, Universidade de São Paulo, Ribeirão Preto, SP, 14040-903, Brazil.*

*^5^Instituto de Química, Universidade Estadual Paulista, Araraquara, SP, 14801-970, Brazil.*

*npelopes@fcfrp.usp.br and [alopes@unaerp.br](mailto:alopes@unaerp.br)

*** Corresponding author.** Tel.: +55 16 36036892; fax: +55 16 36037030

**Figure S1.** Calibration curve of maytenin and 22β-hydroxy-maytenin.

**Table S1.** ^13^C NMR data of 22β-hydroxy-maytenin isolated from *P. laevigata* after incorporation of 1-^13^C-D-glucose (CDCl_3_, 25°C).

| **C** | δ^b^ | δ | **Relative**  **intensity of signal** | | **ΔC = 1,1% x M**  **N** |
| --- | --- | --- | --- | --- | --- |
|  |  |  | **M** | **N** |  |
| **1** | *120.2* | *119.8* | 1.8 | 0.4 | **5.0** |
| **2** | *178.8* | *178.4* | 0.4 | 0.3 | **1.5** |
| **3** | *146.5* | *146.0* | 1.9 | 0.4 | **5.2** |
| **4** | *117.6* | *117.3* | 0.5 | 0.4 | **1.4** |
| **5** | *128.2* | *127.7* | 2.1 | 0.5 | **4.6** |
| **6^a^** | *134.1* | *133.8* | 0.5 | 0.5 | **1.1** |
| **7** | *118.6* | *118.1* | 1.8 | 0.5 | **4.0** |
| **8** | *168.8* | *168.5* | 0.5 | 0.4 | **1.4** |
| **9** | *43.0* | *42.6* | 2.7 | 0.6 | **5.0** |
| **10** | *165.1* | *164.7* | 0.6 | 0.5 | **1.3** |
| **11** | *34.4* | *34.0* | 0.5 | 0.5 | **1.1** |
| **12** | *30.4* | *29.9* | 0.5 | 0.5 | **1.1** |
| **13** | *41.0* | *40.6* | 2.6 | 0.8 | **3.6** |
| **14** | *44.7* | *44.3* | 0.8 | 0.7 | **1.3** |
| **15** | *28.7* | *28.3* | 1.5 | 0.5 | **3.3** |
| **16** | *29.9* | *29.5* | 0.5 | 0.5 | **1.1** |
| **17** | *45.2* | *44.8* | 1.0 | 0.8 | **1.4** |
| **18** | *45.5* | *45.0* | 2.0 | 0.7 | **3.1** |
| **19** | *32.4* | *32.0* | 1.7 | 0.5 | **3.7** |
| **20** | *41.3* | *40.9* | 0.6 | 0.6 | **1.1** |
| **21** | *213.7* | *213.6* | 0.7 | 0.6 | **1.3** |
| **22** | *76.8* | *76.4* | 2.2 | 0.5 | **4.8** |
| **23** | *10.7* | *10.3* | 4.0 | 0.7 | **6.3** |
| **25** | *39.6* | *39.1* | 2.1 | 0.5 | **4.6** |
| **26** | *22.0* | *21.6* | 3.1 | 0.6 | **5.7** |
| **27** | *20.9* | *20.5* | 1.8 | 0.5 | **4.0** |
| **28** | *25.4* | *25.0* | 2.2 | 0.5 | **4.8** |
| **30** | *15.1* | *14.7* | 3.0 | 0.8 | **4.1** |

U: control experiments with unlabeled precursor; L: labeling experiment with ^13^C precursor; ΔC: increase in the relative intensity (significant increases in bold for enriched carbons).^a^Value used as reference. ^b^Literature values to 22β-hydroxy-maytenin (Takaishi et al., 1997).

**(b)**

**(a)**

**Figure S2.** ^13^C NMR (125 MHz, CDCl_3_) spectra of **2** with natural isotopic abundance **(a)** and **2** after feeding experiments with 1-^13^C-D-glucose **(b)**.

**Figure S3.** Plausible biosynthetic pathway of quinonemethide triterpenoids (**1** and **2**) proceeds by MVA pathway from 1-^13^C-D-glucose metabolism.

**
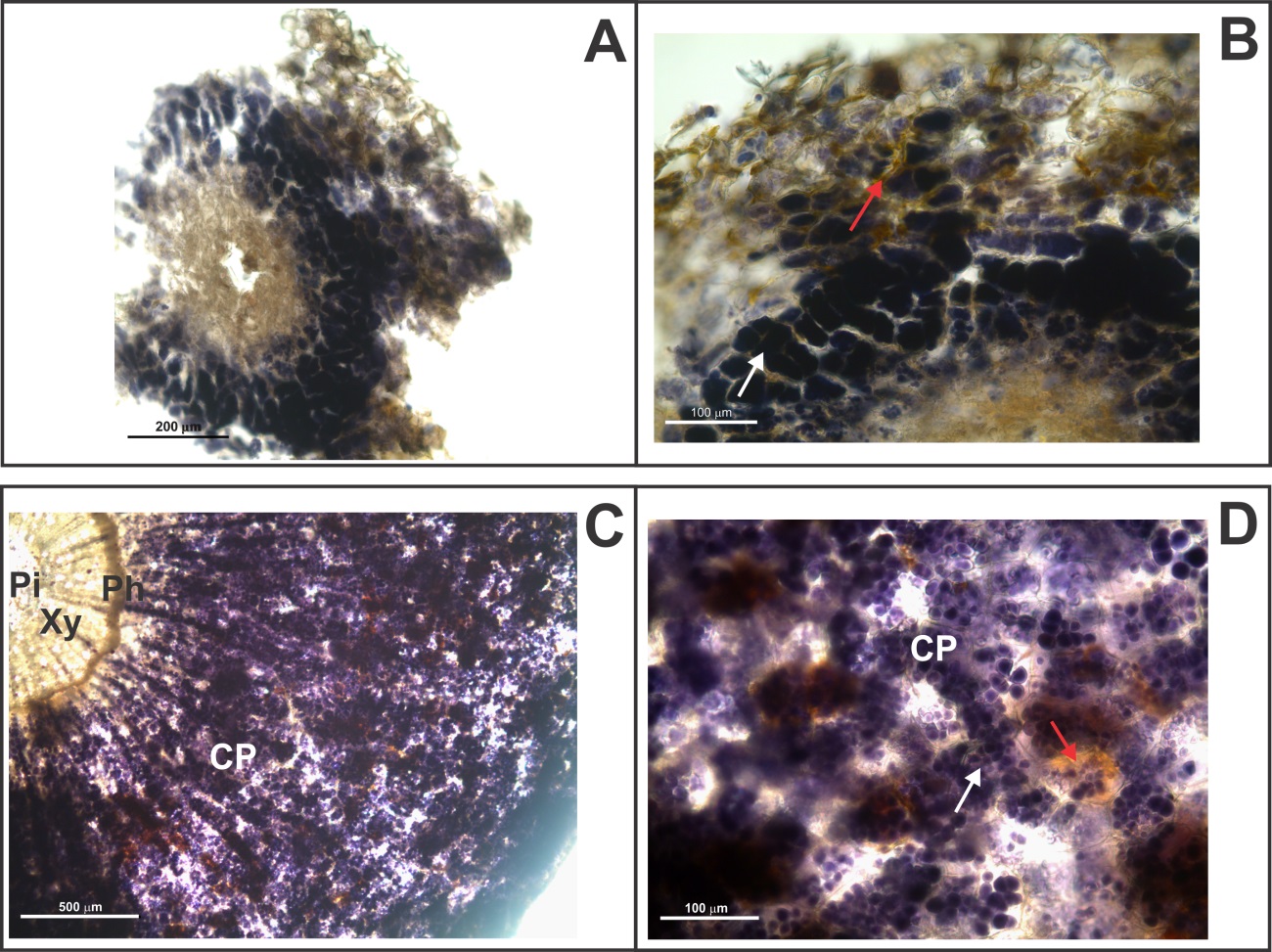
**

**Figure S4.** Histochemical analyses of culture root (*in vitro*) [A and B] and *in situ* [C and D] of *P. laevigata* stained with lugol dye. Detail showing substance(s) with intense orange colour (red arrow) and starch grains in black (white arrow). CP: cortical parenchyma; Ph: phloem; Pi: pitch; Xy: xylem.

**
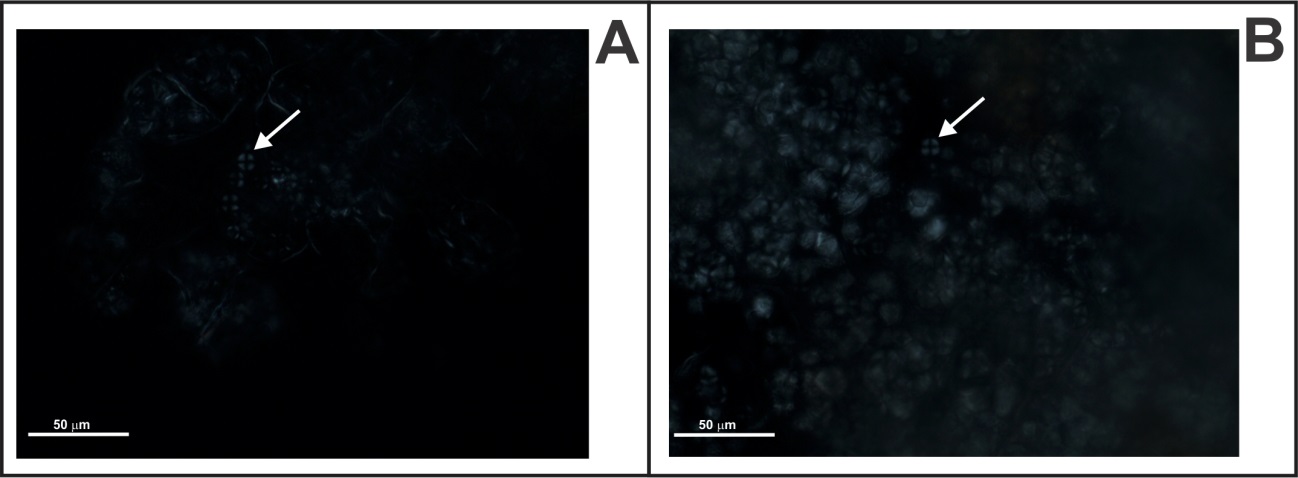
Figure S5.** Transversal sections of culture root (*in vitro*) [A] and *ex vitro* [B] of *P. laevigata* observed with polarized light. Note the presence of starch grains in the cortical parenchymatic cells (white arrow).


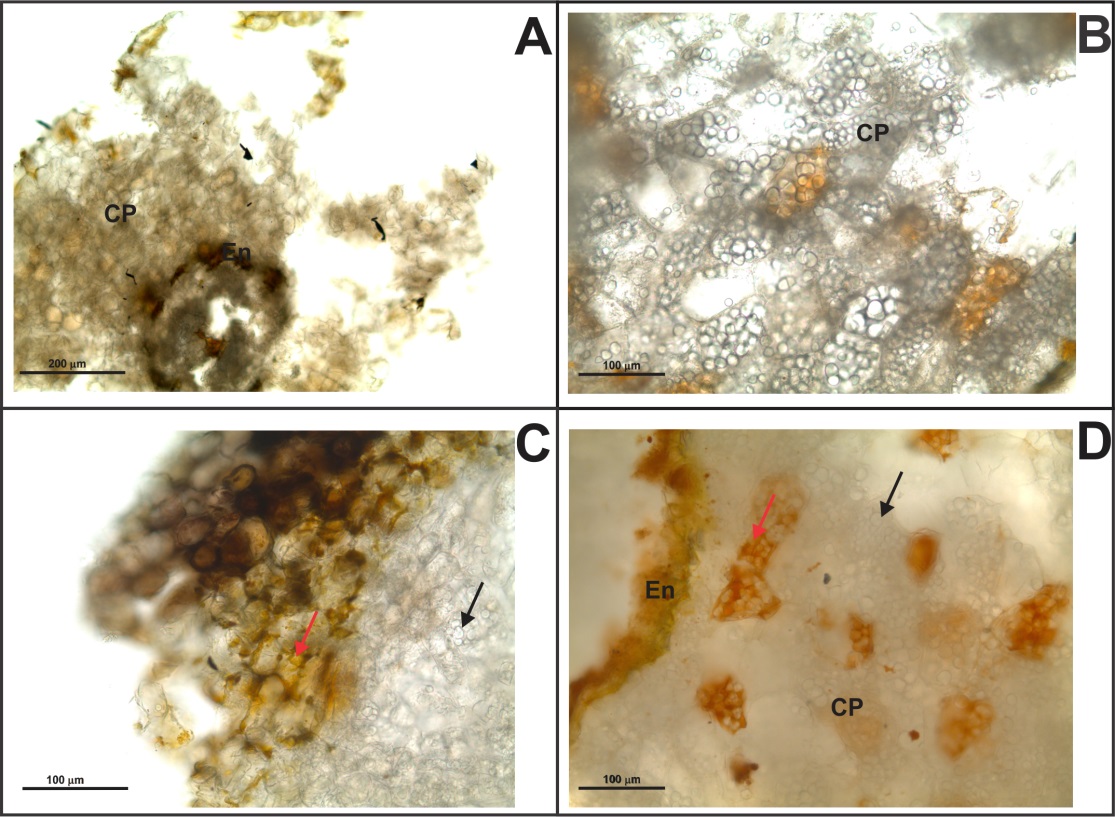


**Figure S6.** No stained transversal sections of culture root (*in vitro*) [A and C] and *in situ* [B and D] of *P. laevigata*. Note the presence of starch grains (black arrow) in the cortical parenchymatic tissue and the substance(s) with intense orange colour (red arrow) in periderm and cortical parenchymatic tissue. CP: cortical parenchyma; En: endoderm.


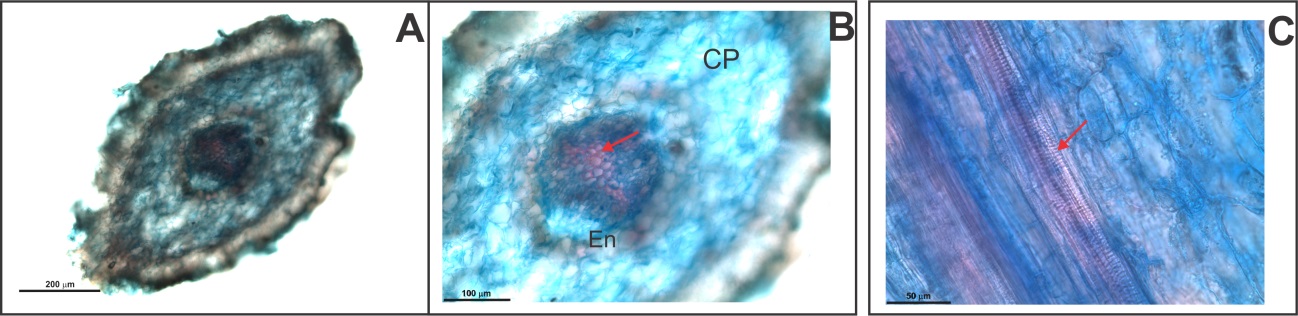


**Figure S7.** Transversal (A and B) and longitudinal sections (C) of *in vitro* root cultures from *P. laevigata*. Dye: safranine/astra blue. Detail of xylematic elements (red arrow). CP: cortical parenchyma; En: endoderm.


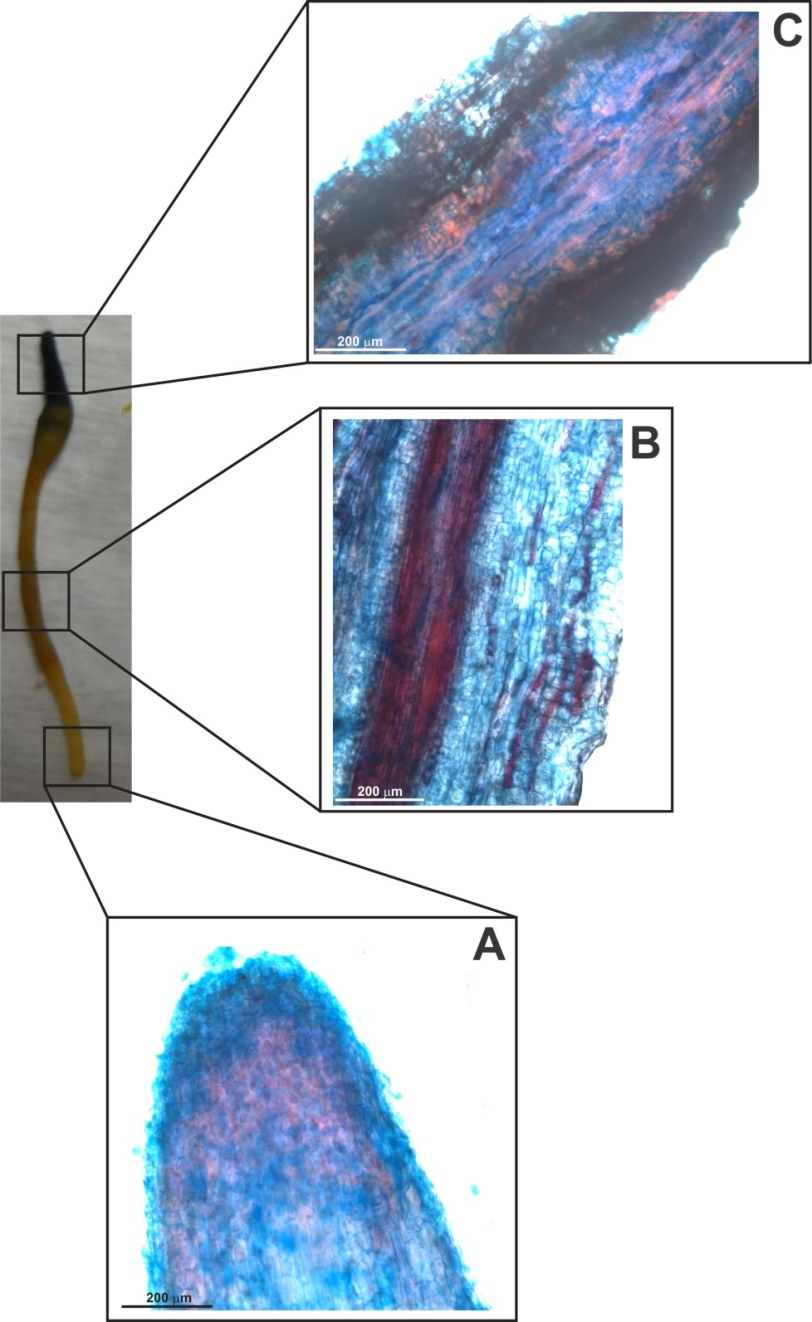


**Figure S8.** Longitudinal sections of *in vitro* root cultures from *P. laevigata* from different regions: near to root cap (A), differentiating region (B) and root primary structure (C). Dye: safranine/astra blue.


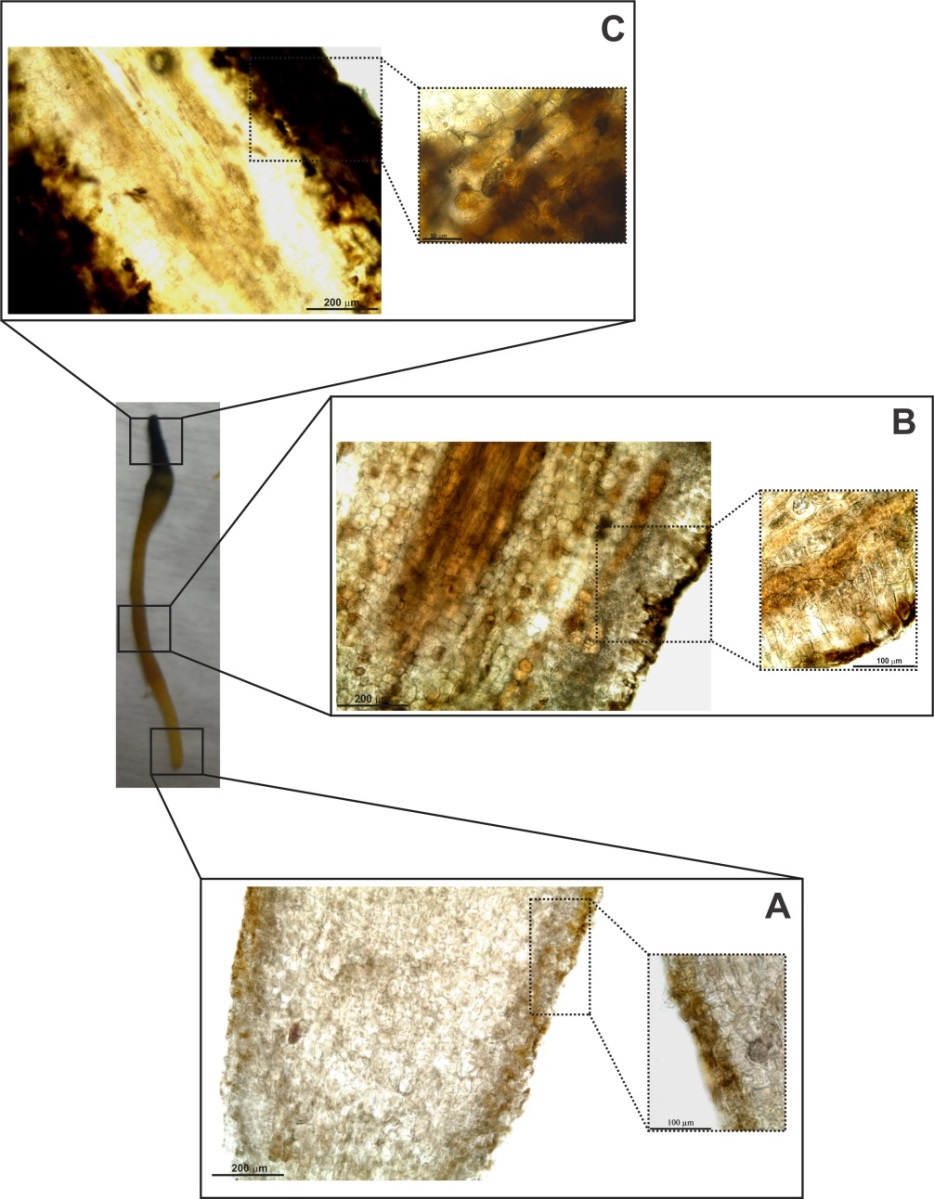


**Figure S9.** Longitudinal sections of *in vitro* root cultures from *P. laevigata* from different regions: root cap (A), differentiating region (B) and root primary structure (C). No dye was applied.

**
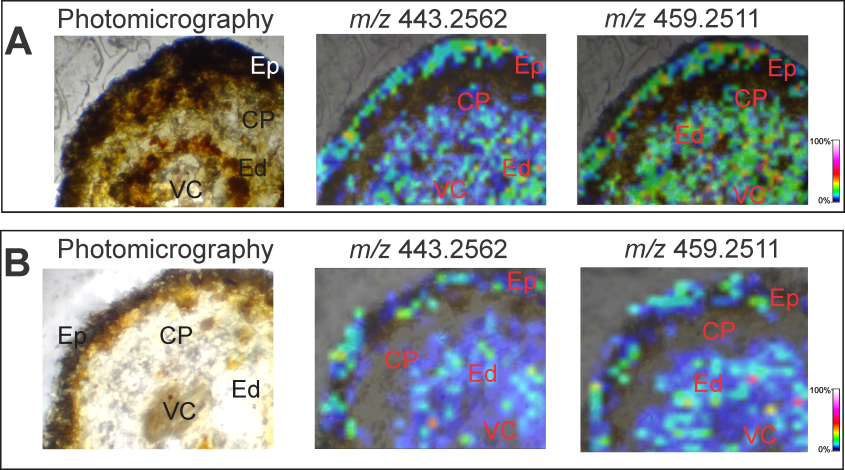
**

**Figure S10.** Transversal sections of root cultures from *Peritassa laevigata* obtained from different parts: differentiating region (**A**, base root) and primary structure (**B*,*** near to root cap). MALDI-MS images were reconstructed with ions *m/z* 443.2562 [M+Na]^+^ and 459.2511 [M+Na]^+^, relative to maytenin **1** and **2**, respectively. CP: cortical parenchyma; Ed: endoderm; Ep: epiderm; VC: vascular cylinder.

**REFERENCES**

Takaishi, Y. *et al.* Triterpenoid inhibitors of interleukin-1 secretion and tumour-promotion from *Tripterygium wilfordii* var. regelii. *Phytochemistry* **45**, 969-974 (1997).
